# Effects of exercise interventions on physical function, cognitive function and quality of life of frail older adults in nursing homes: a systematic review and meta-analysis

**DOI:** 10.3389/fpsyg.2025.1679734

**Published:** 2025-09-05

**Authors:** Yingbo Zhu, Yu Zhang, Xiao Li, Zhijuan Du

**Affiliations:** ^1^School of Physical Education, Physical Education College of Henan University, Kaifeng, China; ^2^School of Physical Education, Physical Education College of Linyi University, Linyi, China

**Keywords:** older adults, nursing facility, exercise, meta-analysis, systematic review

## Abstract

**Objective:**

Older adults often face frailty as they age, characterized by a decline in physical and mental health, leading to increased reliance on caregiving services, particularly in nursing homes. Exercise interventions have emerged as an effective means of improving health outcomes, but their efficacy for this population remains unclear. The aim of this study was to investigate the effects of exercise interventions on physical function, cognitive function and quality of life of frail older people in a nursing facility.

**Methods:**

Data were systematically collected from five major databases and analyzed using RevMan 5.4 and Stata 17.0. Standardized mean differences (SMD) with 95% confidence intervals were calculated to evaluate health outcomes—including physical function, cognitive function, and quality of life—among frail older adults, and heterogeneity was assessed using the *I*^2^ statistic.

**Results:**

A total of 16 studies comprising 1,444 participants (mean age ranging from 73.3 ± 6.4 to 87.3 ± 5.3 years) were included in the meta-analysis. The findings consistently demonstrated that exercise interventions produced significant improvements across multiple domains, including physical performance [*SMD* = 0.54, 95% CI (0.38, 0.70), *p* < 0.001], mobility [*SMD* = −2.42, 95% CI (−3.96, −0.87), *Z* = 3.07, *p* < 0.05], muscle strength [*SMD* = 2.00, 95% CI [0.32, 3.68], *p* < 0.05], quality of life [*SMD* = 1.94, 95% CI (0.37, 3.51), *p* < 0.05], and cognitive function [*SMD* = 0.64, 95% CI (0.13, 1.15), *p* < 0.05]. Notably, physical activity yielded pronounced benefits in alleviating depression [*SMD* = −0.78, 95% CI (−1.07, −0.49), *p* < 0.001] and frailty [*SMD* = −1.44, 95% CI (−1.74, −1.15), *p* < 0.001].

**Conclusion:**

This study demonstrated that exercise interventions significantly improve physical function, mobility, grip strength, and lower limb strength. The results also showed positive effects on alleviating depressive symptoms and improving frailty status. With effect sizes indicating a substantial impact. Furthermore, subgroup analysis revealed that a regimen of at least three sessions per week, each lasting no less than 40 min, was associated with the most favorable outcomes.

**Systematic review registration:**

https://www.crd.york.ac.uk/PROSPERO/view/CRD42024614885, ID: 614537.

## 1 Introduction

The world is experiencing a significant trend of population aging, accompanied by an increase in age-related health issues such as non-communicable diseases and functional disabilities, leading to a substantial rise in global care demands (Izquierdo et al., 2021). Research predicts that the number of older people requiring care will increase globally from 101 million in 2010 to 277 million in 2050 (Reber et al., 2020). Against this backdrop, nursing facility residents represent a rapidly growing and highly vulnerable population. Studies indicate that up to 50% of nursing home residents suffer from frailty, a condition that significantly impairs their quality of life and functional independence (Dent et al., 2019a; Kojima, 2015). Frailty is a complex clinical syndrome closely associated with aging, characterized by a decline in the physiological reserve of multiple organ systems and often accompanied by progressive cognitive deterioration, ranging from mild cognitive impairment to severe dementia (Clegg et al., 2013). Importantly, frailty is dynamic and potentially reversible, existing on a continuum from robustness to frailty, and is considered modifiable during its progression (Dent et al., 2019a). Consequently, functional decline related to frailty is regarded as a potentially preventable disability, emphasizing the importance of early identification and intervention (Dent et al., 2019b).

Currently, interventions for frailty symptoms primarily focus on strategies such as nutritional supplementation and pharmacological treatments (Srinivas-Shankar et al., 2010; Tieland et al., 2012). However, existing research suggests that these approaches have limited efficacy in improving frailty status. For example, the effect of protein on muscle mass in sedentary older adults is not yet significant, and its impact on muscle strength and physical performance shows variability (Zhang et al., 2025). Functional decline in the elderly is often associated with metabolic dysregulation, particularly hyperinsulinemia, which may impair the utilization of amino acids in muscle tissue, thereby reducing protein synthesis capacity (Ferreira et al., 2018; Volpi et al., 2000). In addition, pharmacological interventions face several challenges, including adverse drug reactions, drug–drug interactions, poor adherence, and inappropriate prescribing. These issues are closely linked to the higher morbidity and mortality observed in frail older adults (Romera-Liebana et al., 2018). Given the limitations of current interventions, there is an urgent need to explore safer, more effective, and widely applicable alternatives.

Since quantitative declines in physical function and independence are core features of frailty, exercise interventions have emerged as a promising, low-cost, and high-safety therapeutic option (Dent et al., 2016; Devereux et al., 2019). A study published in The Lancet reported that both single-modality and multicomponent exercise programs improved muscle strength, balance, and mobility in frail older individuals to varying degrees (Dent et al., 2019a). Furthermore, a 12-week intervention conducted by Langlois et al. (2013), which combined aerobic and resistance training, significantly improved the quality of life, cognitive function, and physical performance in frail older adults. These studies provide strong evidence for the significant effects of exercise interventions in alleviating frailty symptoms. However, residents in long-term care nursing homes typically suffer from multiple chronic conditions, functional impairments, severe cognitive deficits, and depression, with concerns about falls and related injuries leading to low levels of physical activity (Arrieta et al., 2018). This situation makes it difficult to implement exercise interventions in nursing homes, and current meta-analyses lack a comprehensive review of this specific population. Only a few meta-analyses have shown that frail older adults living in the community can achieve improvements in muscle strength through multi-component exercise interventions (*MD* = 2.46, *p* = 0.007) (Li et al., 2023). Notably, the frailty prevalence among nursing home residents (approximately 50%) is much higher than that in the community elderly population (approximately 10%) (Dent et al., 2019a; Kojima, 2015). To date, meta-analytic evidence on exercise interventions for frail nursing home residents remains scarce, and the optimal exercise modes and parameters are still unclear.

Therefore, this study aims to systematically evaluate the effects of exercise interventions (either alone or in combination with other methods) on physical function, cognitive function, and quality of life in frail older adults in nursing homes, providing theoretical support and practical guidance for developing more effective and evidence-based frailty management strategies and exercise intervention programs.

## 2 Materials and methods

This systematic evaluation and meta-analysis was conducted in accordance with the Preferred Reporting Items for Systematic Evaluation (PRISMA) guidelines and meta-analysis statement (Moher et al., 2014). This study is registered with the International Prospective Systems Evaluation Registry (PROSPERO): registration number CRD42024614885.

### 2.1 Search strategy

Literature searches were performed across five databases (PubMed, Embase, Web of Science, Cochrane Library, and CINAHL) from their inception to May 2025. The search strategy utilized a combination of Medical Subject Headings (MeSH) terms and free words, with no language restrictions applied. Additionally, preprint literature, references from screened studies, and previous systematic reviews were examined to identify further relevant studies. Detailed search strategies are provided in Supplementary Table S1.

### 2.2 Eligibility criteria

The study selection criteria were formulated using the Population, Intervention, Comparison, Outcome, and Study Design (PICOS) framework. (1) Population: adults ≥ 65 years of age residing in an institutionalized setting (e.g., nursing home or long-term care facility) with a confirmed diagnosis of frailty or pre-frailty status. Frailty was defined using the Fried frailty phenotype (Fried et al., 2001), in which individuals meeting three or more of the five criteria were classified as frail, while those meeting one or two criteria were classified as pre-frail. (2) Intervention: The experimental group received physical training as an intervention. (3) Comparison: The control group received standard care. (4) Outcomes: Primary endpoints included indicators of physical functioning, cognitive functioning, and quality of life. (5) Study Design: Randomized controlled trials (RCTs) or quasi-experimental studies published in English. Studies were excluded if they met any of the following criteria: (1) lack of full-text availability; (2) inadequate descriptions of interventions or insufficient statistical data for analysis; (3) categorization as case reports, conference abstracts, systematic reviews, or meta-analyses; and (4) inclusion of participants with major medical conditions such as severe heart disease.

### 2.3 Study selection and data extraction

This study was conducted by two researchers who independently carried out the systematic literature screening and data extraction process. Initially, titles and abstracts were screened to exclude unrelated studies, duplicate reports from the same randomized controlled trials, and animal studies. Then, the full texts of the remaining studies were reviewed and systematically assessed according to the pre-defined inclusion and exclusion criteria. The researchers independently extracted the following data from each study: basic study information (authors, publication year, and location), participant characteristics (sample size, gender, mean age, and frailty assessment criteria), details of the intervention (type, duration, frequency, and intensity of exercise), and the intervention plan for the control group. For multi-arm trials, only data from the relevant exercise groups were extracted. All data were recorded in a standardized table format. If there were discrepancies between the two researchers’ data extraction, a third researcher would discuss the differences, and a consensus would be reached to resolve them. The entire process strictly followed the pre-set criteria and maintained detailed records to ensure the methodological validity and reliability.

### 2.4 Outcome measures

The primary outcome indicators in this study include physical functioning, cognitive functioning and quality of life. Age-related declines in physical functioning are manifested in three main areas: physical performance, mobility and muscular strength. Physical performance was assessed using the Short Physical Performance Battery (SPPB), mobility was evaluated using the Timed Up and Go (TUG) test, and muscular strength was measured through grip strength and lower limb strength (Farinatti et al., 2013; Zhang et al., 2020). For assessing lower limb strength specifically, two instruments with similar assessment methods and indicators were used: the chair stand test (STS-5) and the five times sit-to-stand test (FTSST) (Farinatti et al., 2013; Zhang et al., 2020). Cognitive function was measured using the Mini Mental State Examination (MMSE) and the Standardized Mini Mental State Examination (SMME). Quality of life was assessed through health-related quality of life indices based on Danish norms, using the EQ-5D scale, and also through the health-related quality of life visual analog scale (EQ-5D VAS).

For the secondary outcome indicators, frailty was assessed using the frailty indicator score reported in the article to determine frailty status. Depression was evaluated using the Geriatric Depression Scale (GDS).

### 2.5 Risk of bias assessment

Two researchers independently assessed the quality of the included studies using the criteria outlined in the Cochrane Handbook for Systematic Reviews of Interventions (n.d.). The evaluation index includes the following seven items: randomization sequence generation, allocation concealment, a blinding method for study subjects and interveners, a blinding method for results measurement, incomplete result data, selective reporting bias and other bias. Each trial was rated as having a “low risk,” “unclear risk,” or “high risk” of bias. A study was classified as being at “high risk of bias” if two or more domains were identified as high risk. Discrepancies between the two researchers regarding the risk of bias were resolved through discussion or consultation with a third researcher.

### 2.6 Statistical analysis

Meta-analyses were conducted using RevMan 5.4 (Cochrane Collaboration, Oxford, UK). Clinical heterogeneity between studies was assessed using chi-squared tests. A fixed-effects model was applied when *p* ≥ 0.1 and *I*^2^ values ranged from 25 to 49%, indicating low heterogeneity, for moderate (*I*^2^ = 50–74%) or high heterogeneity (*I*^2^ > 75%) with *p* < 0.1, a random-effects model was used. Continuous data were analyzed using either standardized mean difference (SMD), and pooled effects were expressed as SMD with 95% confidence intervals (Cl). For studies with high heterogeneity (α = 0.05), each study was excluded one by one and the meta-analysis was rerun. The differences in results before and after excluding each study was compared to assess its impact on the overall results and confirm the stability of the findings. Additionally, a descriptive analysis was conducted for the studies with high heterogeneity to provide further information for the heterogeneity analysis. Publication bias was evaluated using Begg’s and Egger’s tests in Stata 17.0. If significant publication bias was detected, the trim-and-fill method was applied to address it, enhancing the robustness and reliability of the results. In addition, subgroup analyses were conducted to explore whether participant characteristics or intervention characteristics influenced between-study effect sizes.

## 3 Results

### 3.1 Study selection

**Figure 1:**
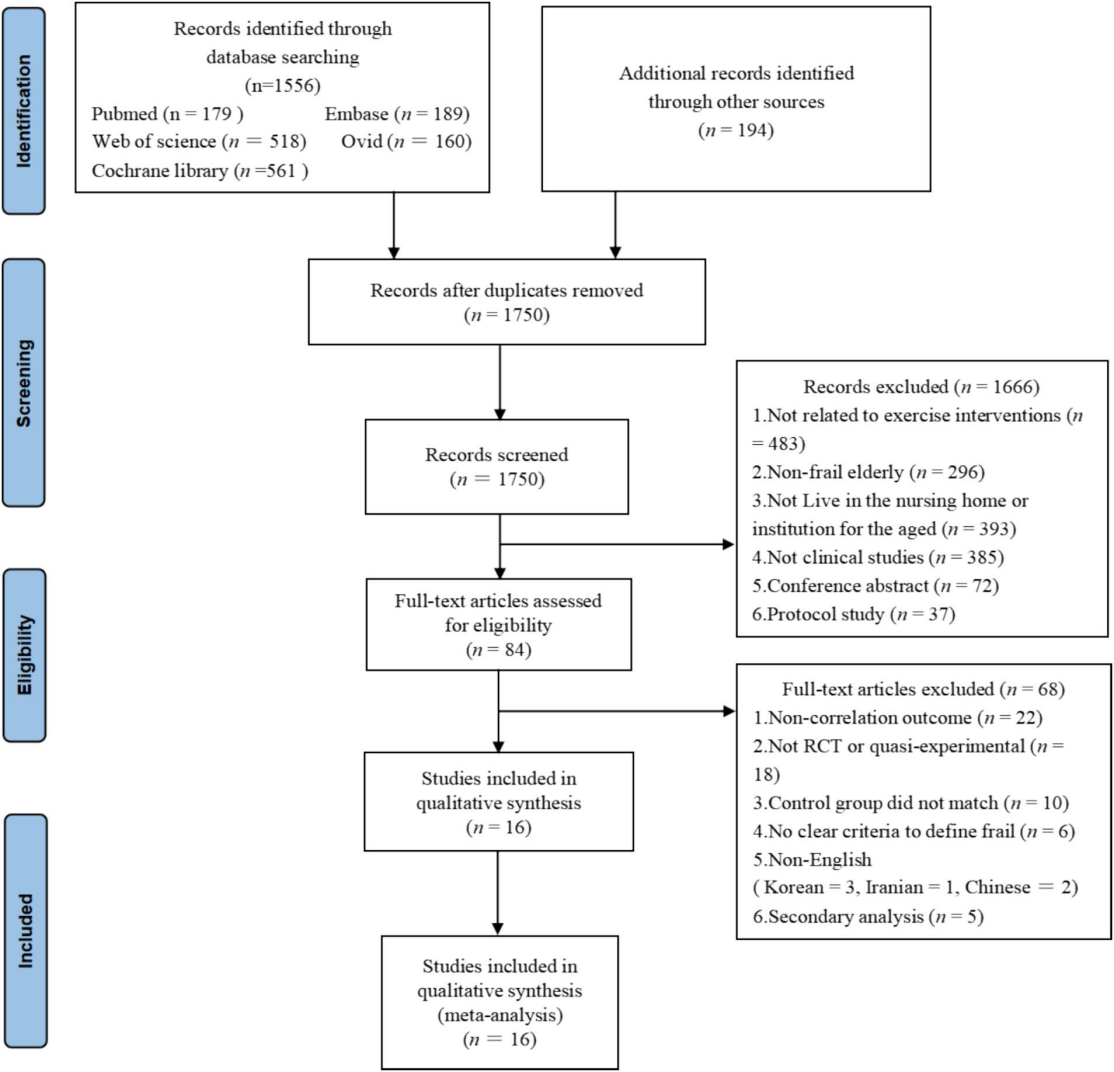
PRISMA flow diagram of the study selection process.

Initially, 1801 studies were identified through five databases. Then, 51 duplicate studies were screened and excluded using EndNote software. The remaining 1750 studies were screened based on study design, title, and abstract, of which 1,666 were excluded because they did not meet the PICOS criteria. The full text of 84 studies was screened and 68 studies were subsequently excluded for the following reasons: does not address the primary outcome indicators examined in this paper (*n* = 21); non-RCT or quasi-experimental studies (*n* = 17); unmatched controls (*n* = 6); no clear criteria to define frailty (*n* = 4); non-English language studies (Chinese = 2, Korean = 3, Iranian = 1); secondary analyses of initial data (*n* = 5); insufficient data for analysis (*n* = 9). Eventually 16 studies were included in our final analysis. The detailed process of study selection in this paper is shown in Figure 1.

### 3.2 Study characteristics

In the end, 16 studies from 9 countries were included: Portugal (*n* = 3), China (*n* = 4), Spain (*n* = 3), Brazil (*n* = 1), UK (*n* = 1), South Korea (*n* = 1), Turkey (*n* = 1), Singapore (*n* = 1) and New Zeal and (*n* = 1). A total of 1,444 participants were included in the studies, 652 in the experimental group and 792 in the control group, with an average age range of 73.3–87.3 years for the participants. Of the study populations, three studies included only pre-frail older adults (Lee, 2024; Romera-Liebana et al., 2018; Tan et al., 2023), nine studies included frail older adults (Apóstolo et al., 2019a; Barros et al., 2024; Chou et al., 2022; García-Gollarte et al., 2023; Liu et al., 2022; Mollinedo Cardalda et al., 2019; Sahin et al., 2018; Swales et al., 2022; Wadsworth et al., 2022), and four studies included both pre-frail and frail older adults (Wadsworth et al., 2022; Apóstolo et al., 2019a), with one study explicitly defining the participants as a cognitively declining population (Chen et al., 2021). Fourteen studies used the Fried frailty criteria (Fried frailty criteria) to identify frailty (Barros et al., 2024; Caldo-Silva et al., 2021; Chen et al., 2021; Chou et al., 2022; Ferreira et al., 2018; García-Gollarte et al., 2023; Lee, 2024; Liu et al., 2022; Mollinedo Cardalda et al., 2019; Romera-Liebana et al., 2018; Sahin et al., 2018; Swales et al., 2022; Tan et al., 2023; Zheng et al., 2025), whereas the other two studies described frailty through functional walking tests or cognitive assessments (Apóstolo et al., 2019a; Wadsworth et al., 2022).

The exercise interventions included in the study were predominantly multicomponent training (e.g., strength training combined with aerobic, flexibility, balance, or resistance training), and other forms of intervention included vibration training (Wadsworth et al., 2022), tai chi (Liu et al., 2022), and acupuncture exercises (Chou et al., 2022). The control group received interventions focusing on routine care, health education and flexibility exercises. The duration of physical exercise ranged from 30 to 60 min per day, with a frequency of 2–7 days per week for 8–48 weeks. Follow-up ranged from 4 to 48 weeks. Treatment adherence was assessed by the percentage of training sessions completed, and participants’ adherence levels ranged from 63 to 95.84% in the included studies. Training venues included long-term care facilities (two studies) (Chou et al., 2022; Wadsworth et al., 2022), social geriatric institutions (four studies) (Ferreira et al., 2018; García-Gollarte et al., 2023; Lee, 2024; Sahin et al., 2018), and nursing homes (remaining studies) (Barros et al., 2024; Caldo-Silva et al., 2021; Chen et al., 2021; Liu et al., 2022; Mollinedo Cardalda et al., 2019; Romera-Liebana et al., 2018; Swales et al., 2022; Tan et al., 2023; Zheng et al., 2025). Professionals were responsible for managing and supervising the training programs: five studies were run by experienced physiotherapists (Chen et al., 2021; García-Gollarte et al., 2023; Lee, 2024; Romera-Liebana et al., 2018; Sahin et al., 2018), three studies were supervised by trained coaches (Chou et al., 2022; Ferreira et al., 2018; Swales et al., 2022), and a further four studies were directed by trained researchers (Tan et al., 2023; Wadsworth et al., 2022; Zheng et al., 2025). In addition, one study had a training program implemented directly by a sports specialist (Barros et al., 2024) and another had a training program designed by a sports specialist and conducted by a healthcare professional (Liu et al., 2022). However, two studies did not provide specific implementation information (Caldo-Silva et al., 2021; Mollinedo Cardalda et al., 2019). Detailed information about the included studies and their characteristics is presented in Table 1.

**Table 1:** Characteristics of the included studies.

Author/year	Participants	Age	Interventions	Adherence levels	Outcome measures
Barros et al. (2024)	Frailty (fried criteria) EG (*n* = 28) CG (*n* = 28)	EG:82.93 ± 6.82 CG:82.76 ± 8.24	EG: Combined resistance and cardio training CG: Usual care (3 sessions/wk. for 12 wks, 50–60 min/session, group-based training)	EG:95.84%	SPPB HGS
Ferreira et al. (2018)	Prefrail and frail (fried criteria) EG (*n* = 13) CG (*n* = 24)	EG:73.3 ± 6.4 CG:77.8 ± 8.0	EG: Multi-component exercise program including: mobility, flexibility, aerobic resistance, strength training CG: Usual care (3 sessions/wks for 12 wks, 40 min/session, group-based training)	EG:61.5%	HGS TUG MMSE STS-5
Chen et al. (2021)	Prefrail and frail (fried criteria) EG (*n* = 29) CG (*n* = 30)	EG:84.59 ± 4.21 CG:84.75 ± 5.41	EG: Ogata exercise program. CG: Health education (3 sessions/wk. for 12 wks, 30 min/session, group-based training)	No information	FTSST TUG BBS GDS-15 SF-12 MCS
Sahin et al. (2018)	Frail (fried criteria) EG (*n* = 16) CG (*n* = 16)	EG:84.5 ± 4.81 CG:85.37 ± 4.7	EG: Single repetition 40% RM resistance workout CG: Maintaining daily life without intervention (3 sessions/wk. for 8 wks, 40 min/session, group-based training)	No information	SPPB·HGS Barthel index WHOQL-OLD
Caldo-Silva et al. (2021)	Prefrail and frail (fried criteria) EG (*n* = 13) CG (*n* = 24)	EG:80 ± 6.1 CG:83.1 ± 5.4	EG: Multicomponent training CG: Usual care (2 sessions/wk. for 16 wks, 60 min/session, group-based training)	No information	FTSST MMSE Physical frailty
Wadsworth et al. (2022)	Frailty (functional ambulation) EG (*n* = 36) CG (*n* = 46)	EG:79.4 ± 1.1 CG:84.3 ± 1.3	EG: Whole body vibration training CG: Usual care (3 sessions/wk. for 16 wks, 60 min/session, group-based training)	EG: 93%	Barthel index QoL
Swales et al. (2022)	Frail (fried criteria) EG (*n* = 6) CG (*n* = 5)	EG:85.83 ± 7.83 CG:86.4 ± 7.2	EG: Resistance training CG: Usual care (3–4 sessions/wks for 6 wks, 50 min/session, group-based training)	EG: >80%	SMME SPPB GDS HADS Knee extension strength
García-Gollarte et al. (2023)	Frailty (fried criteria) EG (*n* = 39) CG (*n* = 34)	EG:86 ± 5.9 CG:87.3 ± 5.3	EG: Ogata movement program CG: Usual care (3 sessions/wks for 24 wks, 60 min/session, group-based training)	EG: 71%	TUG BBS SPPB STS-5 Handgrip strength 6MWT
Romera-Liebana et al. (2018)	Prefrail (fried criteria) EG (*n* = 176) CG (*n* = 176)	EG:77.4 ± 7.7 CG:77.2 ± 6.8	EG: Comprehensive interventions (aerobic exercise, nutritional supplementation, memory tasks) CG: Usual care (2 sessions/wks for 6 wks, 60 min/session, group-based training)	No information	SPPB
Tan et al. (2023)	Prefrail (fried criteria) EG (*n* = 80) CG (*n* = 187)	EG:73.39 ± 5.2 CG:71.69 ± 4.99	EG: Multi-component interventions (aerobic Training, resistance training, dual duty, balance training) CG: Health education (2 sessions/wks for 24 wks, 60 min/session, group-based training)	No information	Moca·HGS·SPPB·STS-5 Frail total EQ-VAS
Chou et al. (2022)	Frail (fried criteria) EG (*n* = 40) CG (*n* = 41)	EG:80.95 ± 7.95 CG:78.41 ± 8.21	EG: VA exercise (phoenix fist, tiger fist, dragon fist, trunk fist, and palm beat) CG: Usual care (3 sessions/wks for 24 wks, 40 min/session, group-based training)	No information	HGS·lung function Upper-limb muscle endurance
Liu et al. (2022)	Frailty (fried criteria) EG (*n* = 67) CG (*n* = 68)	EG:80.75 ± 2.99 CG:80.74 ± 2.82	EG: Tai Chi exercise CG: Usual care (5 sessions/wks for 48 wks, 40 min/session, group-based training)	EG: 86.5%	QOL MMSW Frailty score
Apóstolo et al. (2019a,b)	Frailty (Barthel score) EG (*n* = 23) CG (*n* = 21)	EG:79.17 ± 7.59 CG:82.04 ± 9.29	EG: Comprehensive exercise interventions CG: Usual care (2 sessions/wk. for 12 wks, 30 min/session, group-based training)	EG: >87%	Moca GDS-10 Gait speed
Lee et al. (2024)	Frailty (fried criteria) EG (*n* = 30) CG (*n* = 30)	EG:85.18 ± 3.14 CG:84.81 ± 2.71	EG: One-legged standing, chair sitting and walking exercises CG: Usual care (2 sessions/wk. for 8 wks, 50 min/session, group-based training)	EG: >95%	TUG STS-5 Gait speed
Zheng et al. (2025)	Frailty (fried criteria) EG (*n* = 33) CG (*n* = 33)	EG:80.88 ± 8.75 CG:79.52 ± 9.61	EG: AR-based rehabilitation for ADLs CG: Usual care (2 sessions/wk. for 12 wks, 45 min/session, group-based training)	EG: >95%	MMSE FP GDS-10
Mollinedo Cardalda et al. (2019)	Frailty (fried criteria) EG (*n* = 23) CG (*n* = 29)	EG:83.76 ± 8.33 CG:85.17 ± 7.38	EG: aerobics training CG: Usual care (2 sessions/wk. for 12 wks, 60 min/session, group-based training)	No information	MMSE STS-5

CG, control group; EX, experimental group; MMSE, Mini Mental State Exam; SMME, Standardized Mini Mental State Examination; BBS, Berg Balance Scale; SPPB, short physical performance battery; TUG, time up and go test; 6MWT, 6-Minute Walk Test; STS-5, Sit to Stand Test-5; FTSST, Five Times Sit to stand Test; GDS-15, Geriatric Depression Scale-15; SF-12MCS, Short Form 12-item health survey Mental Component Summary; Moca, Montreal Cognitive Assessment; HGS, Handgrip Strength; EQ-VAS, EuroQol-Visual Analog Scale; WHOQL-OLD, WHO Quality of Life-Olde; HADS, Hospital Anxiety and Depression Scale; EURO-QoL, Qol, quality of life.

### 3.3 Quality assessment

**Figure 2:**
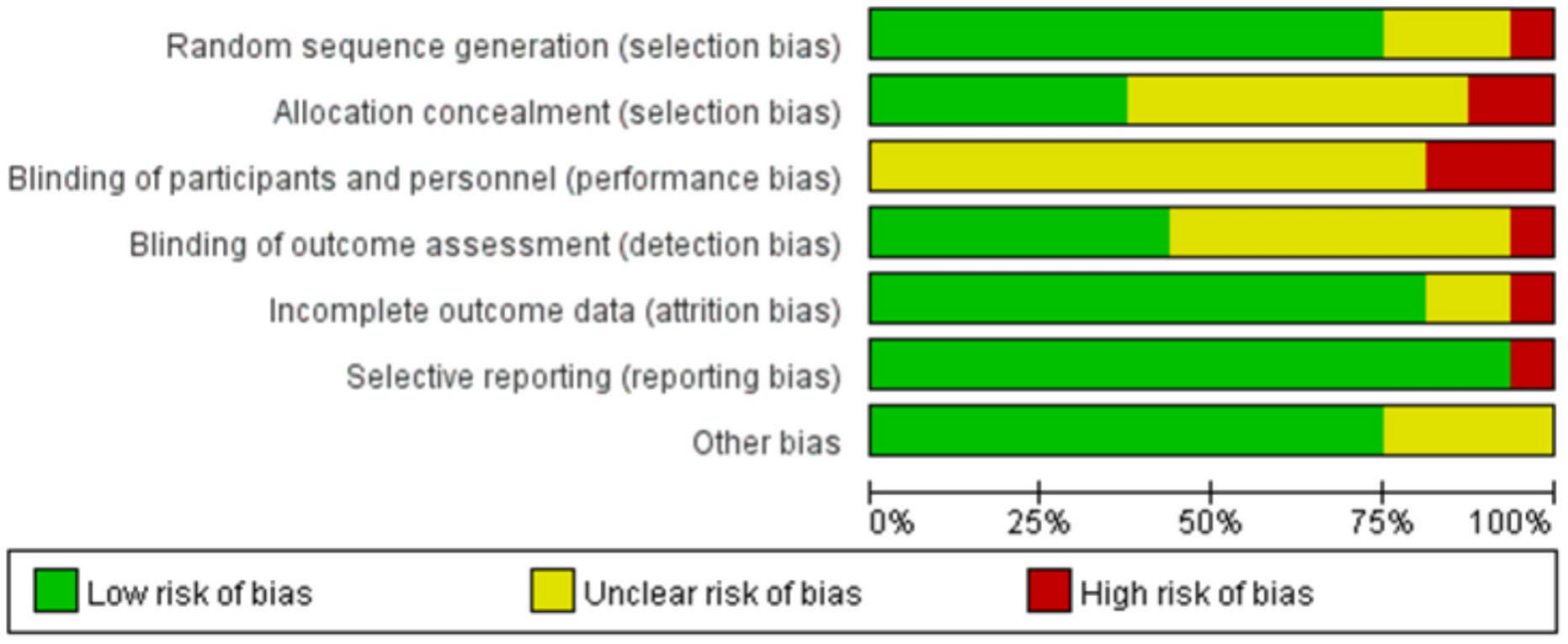
Risk of bias in the included studies.

**Figure 3:**
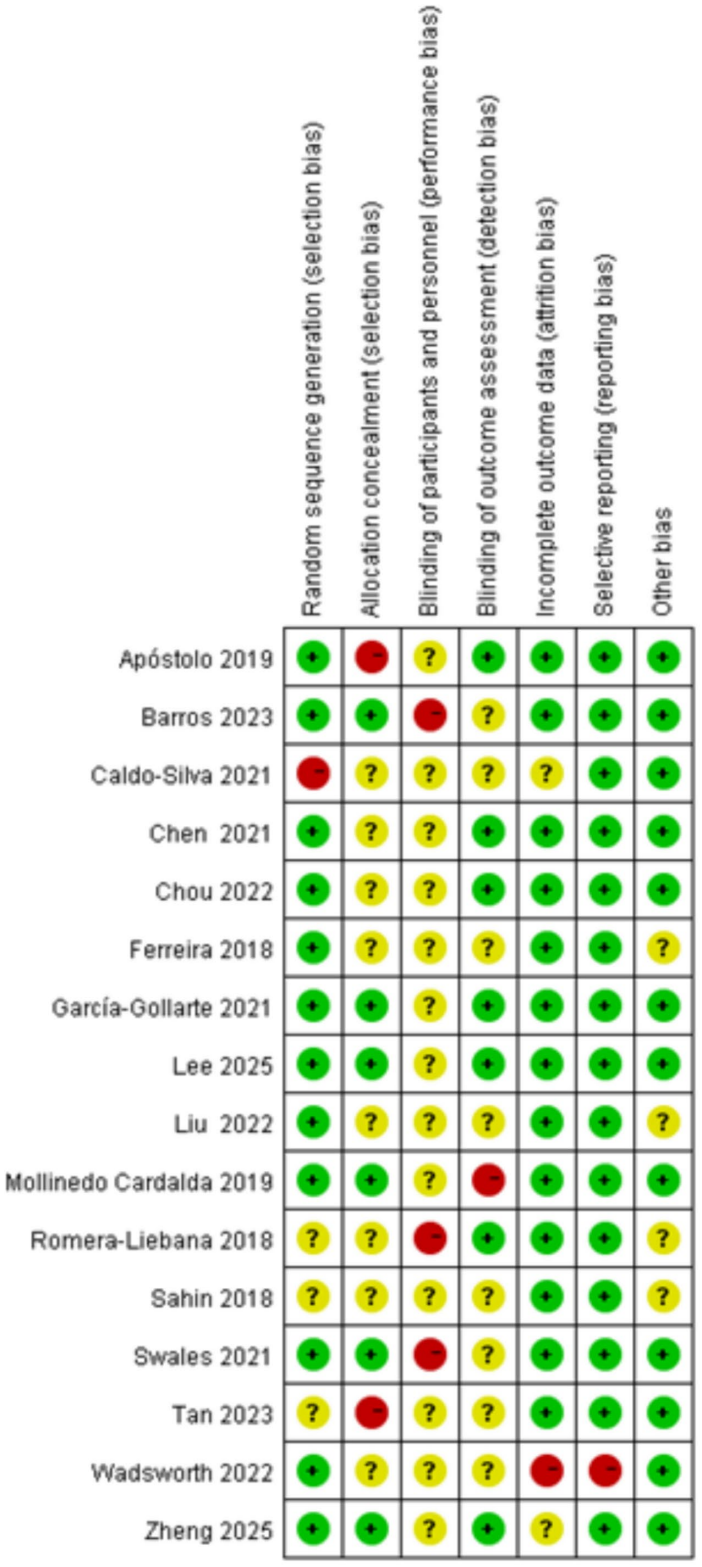
Risk of bias summary in the included studies.

Figures 2, 3 summarize the risk of bias assessments for the included studies. Twelve studies (75%) used simple randomization (e.g., random number tables or computer-generated random numbers) to generate random sequences and were therefore rated at low risk of bias; of the other four studies, three were rated as having an uncertain risk of bias because they did not provide detailed information about the randomized allocation (Sahin et al., 2018; Swales et al., 2022; Tan et al., 2023), and the one that did not use randomized allocation was rated at high risk of bias (García-Gollarte et al., 2023). Six studies (38%) implemented adequate allocation concealment through a third party (Barros et al., 2024; García-Gollarte et al., 2023; Lee, 2024; Mollinedo Cardalda et al., 2019; Swales et al., 2022; Zheng et al., 2025), and seven studies (46%) used single-blind assessment methods (assessor-blind methods) (Apóstolo et al., 2019a; Chen et al., 2021; Chou et al., 2022; García-Gollarte et al., 2023; Lee, 2024; Romera-Liebana et al., 2018; Zheng et al., 2025). Twelve studies reported all outcomes in full, making selective reporting less likely. In terms of other biases, all but four studies (Ferreira et al., 2018; Romera-Liebana et al., 2018; Sahin et al., 2018; Swales et al., 2022) provided detailed reporting of baseline assessments, interventions, outcomes, and duration of follow-up, and were therefore rated at low risk of bias. However, due to technical limitations, the included studies were unable to blind participants and therapists. Overall, the majority of included studies demonstrated a low or moderate risk of major bias.

### 3.4 Effects on both the main and secondary outcomes

#### 3.4.1 Physical function

##### 3.4.1.1 Physical performance

**Figure 4:**
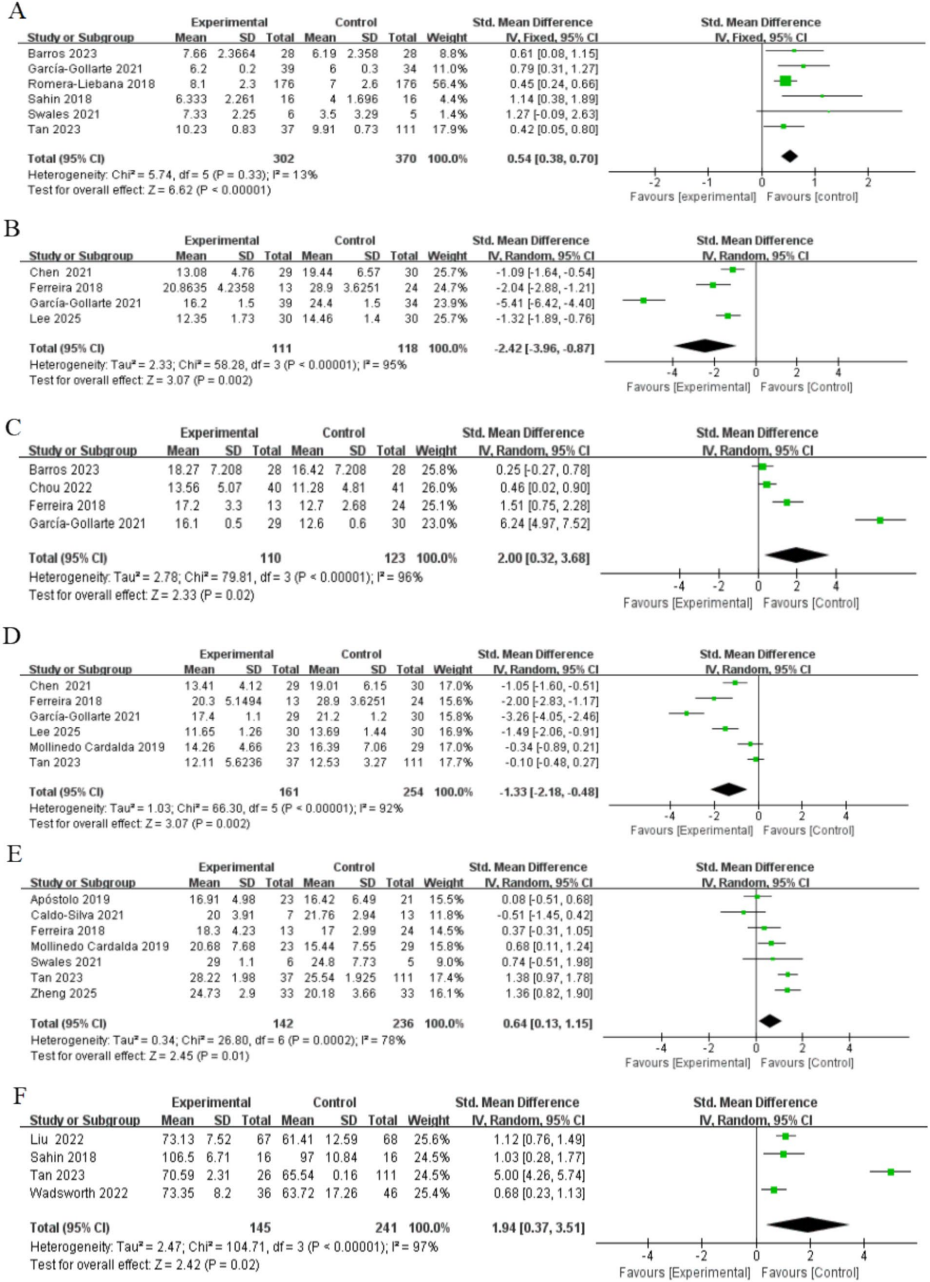
Forest plots of main outcomes. **(A)** Physical performance; **(B)** mobility; **(C)** handgrip strength; **(D)** lower limb strength; **(E)** Cognitive ability; **(F)** Quality of life. Horizontal lines, 95% CIs of each study; green squares, SMDs of each study (size represents the weight given to the study in the meta-analysis): diamond, summary estimate; solid vertical line, null value.

Six studies, involving a total of 672 participants assessed changes in physical performance as measured by the SPPB after intervention completion. Of the included studies, four studies targeted frail older adults (Barros et al., 2024; García-Gollarte et al., 2023; Sahin et al., 2018; Swales et al., 2022) and two studies targeted pre-frail older adults (Romera-Liebana et al., 2018; Tan et al., 2023). The heterogeneity test showed that the heterogeneity between studies was not significant (*I*^2^ = 13%, *p* > 0.05), so the fixed-effects model was used to combine the effect values. The results showed that the exercise intervention significantly improved the SPPB scores of frail older adults in nursing homes [*SMD* = 0.54, 95% CI (0.38, 0.70), *Z* = 6.62, *p* < 0.001] (Figure 4). The Egger’s test indicated the presence of a publication bias (*p* < 0.05), i.e., studies with a larger positive effect value were more likely to be published. After correction by the truncated tail filling method, the effect value decreased slightly [*SMD* = 0.459, 95% CI (0.314, 0.605)], but was still statistically significant, suggesting that the results have some robustness. More details are given in Table 2.

**Table 2:** Meta-analysis of outcome indicators included in the study.

Outcome indicator	Study detail	Effect measure	*p* value	Heterogeneity	Publications bias		
	Studies (*n*)	*SMD*/*MD*/*RR* (95%CI)		*I*^2^ (%)	Egger *P* value	Trim-and-fill Imputed studies	Trim-and-fill *SMD*/*MD*/*RR* (95%)
Physical performance	6	0.54 (0.38, 0.70)	<0.001	13	0.022	3	0.459 (0.314, 0.605)
Mobility	4	−2.42 (−3.96, −0.87)	<0.001	95	0.093	0	–
Muscle power							
Grip strength	4	2.05 (0.39, 3.71)	<0.001	96	0.058	0	–
Lower limb strength	6	−1.33 (−2.18, −0.48)	<0.001	92	0.027	0	−1.33 (−2.18, −0.48)-
Cognitive function	7	0.64 (0.13, 1.15)	0.01	78	0.117	0	–
Quality of life	4	1.84 (0.35, 3.33)	0.02	97	0.39	0	–
Depression	4	−0.78 (−1.07, 0.49)	<0.001	0	0.914	0	–
Frailty	4	−1.44 (−1.74, −1.15)	<0.001	0	0.397	0	–

##### 3.4.1.2 Mobility

Four studies, involving a total of 229 participants, assessed changes in mobility as measured by the TUG after intervention completion. Two of these studies included both frail and pre-frail older adults (Chen et al., 2021; Ferreira et al., 2018), and the other two targeted frail and pre-frail older adults, respectively (García-Gollarte et al., 2023; Lee, 2024). Heterogeneity tests showed high heterogeneity (*I*^2^ = 95%, *p* < 0.001), so effect values were combined using a random effects model. The results showed that the exercise intervention significantly improved exercise capacity in older adults [*SMD* = −2.42, 95% CI (−3.96, −0.87), *Z* = 3.07, *p* < 0.05] (Figure 4). In addition, Egger’s test did not detect significant publication bias (*p* > 0.05), as shown in Table 2.

##### 3.4.1.3 Grip strength

Four studies, involving a total of 233 participants, assessed changes in muscle strength as measured by grip strength testing after completion of the intervention. Three of the studies targeted frail older adults (Barros et al., 2024; Chou et al., 2022; García-Gollarte et al., 2023) and one study included both frail and pre-frail older adults (Ferreira et al., 2018). Heterogeneity tests showed high heterogeneity (*I*^2^ = 96%, *p* < 0.001), so a random effects model was used to combine the effect values. The results showed that the exercise intervention significantly improved muscle strength in older adults [*SMD* = 2.00, 95% CI [0.32, 3.68], *Z* = 2.33, *p* < 0.05] (Figure 4). In addition, Egger’s test did not detect significant publication bias (*p* > 0.05), as shown in Table 2.

##### 3.4.1.4 Lower extremity strength

Six studies, involving a total of 415 participants, assessed lower extremity muscle strength. Five of these studies used the STS-5 to evaluate strength (Ferreira et al., 2018; García-Gollarte et al., 2023; Lee, 2024; Mollinedo Cardalda et al., 2019; Tan et al., 2023), while one study utilized the FTSST (Chen et al., 2021). Participants included frail and pre-frail older adults, with three studies including both groups (Chen et al., 2021; Ferreira et al., 2018; Zheng et al., 2025), two study targeting pre-frail older adults (Lee, 2024; Tan et al., 2023) and the other targeting frail older adults only (García-Gollarte et al., 2023). Heterogeneity tests showed high inter-study heterogeneity (*I*^2^ = 95%, *p* < 0.001), so a random effects model was used to combine the effect values. The results showed that the exercise intervention significantly improved lower limb muscle strength in older adults (*SMD* = −1.33, 95% CI [−2.18, −0.48], *Z* = 3.07, *P* < 0.05) (Figure 4). In addition, as shown in Table 2, the Egger’s test indicated a potential publication bias (*P* < 0.05). However, the results remained stable after applying the trim-and-fill method, suggesting that publication bias was unlikely to have influenced the findings.

#### 3.4.2 Cognitive functioning

Seven studies, involving a total of 378 participants, were included to assess the effects of exercise interventions on cognitive function in older adults. Four studies used the Mini-Mental State Examination (Caldo-Silva et al., 2021; Ferreira et al., 2018; Mollinedo Cardalda et al., 2019; Zheng et al., 2025), two studies used the Moca (Apóstolo et al., 2019b; Tan et al., 2023), and one study used the Standardized Mini-Mental Examination (Swales et al., 2022). The study population consisted of frail and pre-frail older adults, with three studies including both groups (Caldo-Silva et al., 2021; Ferreira et al., 2018; Zheng et al., 2025), three for frail older adults only (Apóstolo et al., 2019b; Mollinedo Cardalda et al., 2019; Swales et al., 2022) and one for pre-frail older adults only (Tan et al., 2023). The heterogeneity test showed high between-study heterogeneity (*I*^2^ = 78%, *p* < 0.001), so the effect values were combined using a random-effects model. The results showed that the exercise intervention did produce a significant enhancement in cognitive function [*SMD* = 0.64, 95% CI (0.13, 1.15), *Z* = 2.45, *p* < 0.05] (Figure 4). In addition, Egger’s test did not detect significant publication bias (*p* > 0.05), as shown in Table 2.

#### 3.4.3 Quality of life

Four studies, involving a total of 386 participants, were included to assess the effect of exercise interventions on the quality of life in older adults. Two studies used the Qol (Liu et al., 2022; Wadsworth et al., 2022) one used the WHOQL-OLD (Sahin et al., 2018) and the other used the EQ-VAS (Tan et al., 2023). The study population consisted of frail and pre-frail older adults, with three studies in frail older adults (Liu et al., 2022; Tan et al., 2023; Wadsworth et al., 2022) and one study in pre-frail older adults (Tan et al., 2023). The heterogeneity test showed high inter-study heterogeneity (*I*^2^ = 97%, *p* < 0.001), so a random effects model was used to combine the effect values. The results showed that the exercise intervention significantly improved the quality of life of older adults [*SMD* = 1.94, 95% CI (0.37, 3.51), *Z* = 2.42, *p* < 0.05] (Figure 4). In addition, Egger’s test did not detect significant publication bias (*p* > 0.05), as shown in Table 2.

#### 3.4.4 Depression

**Figure 5:**
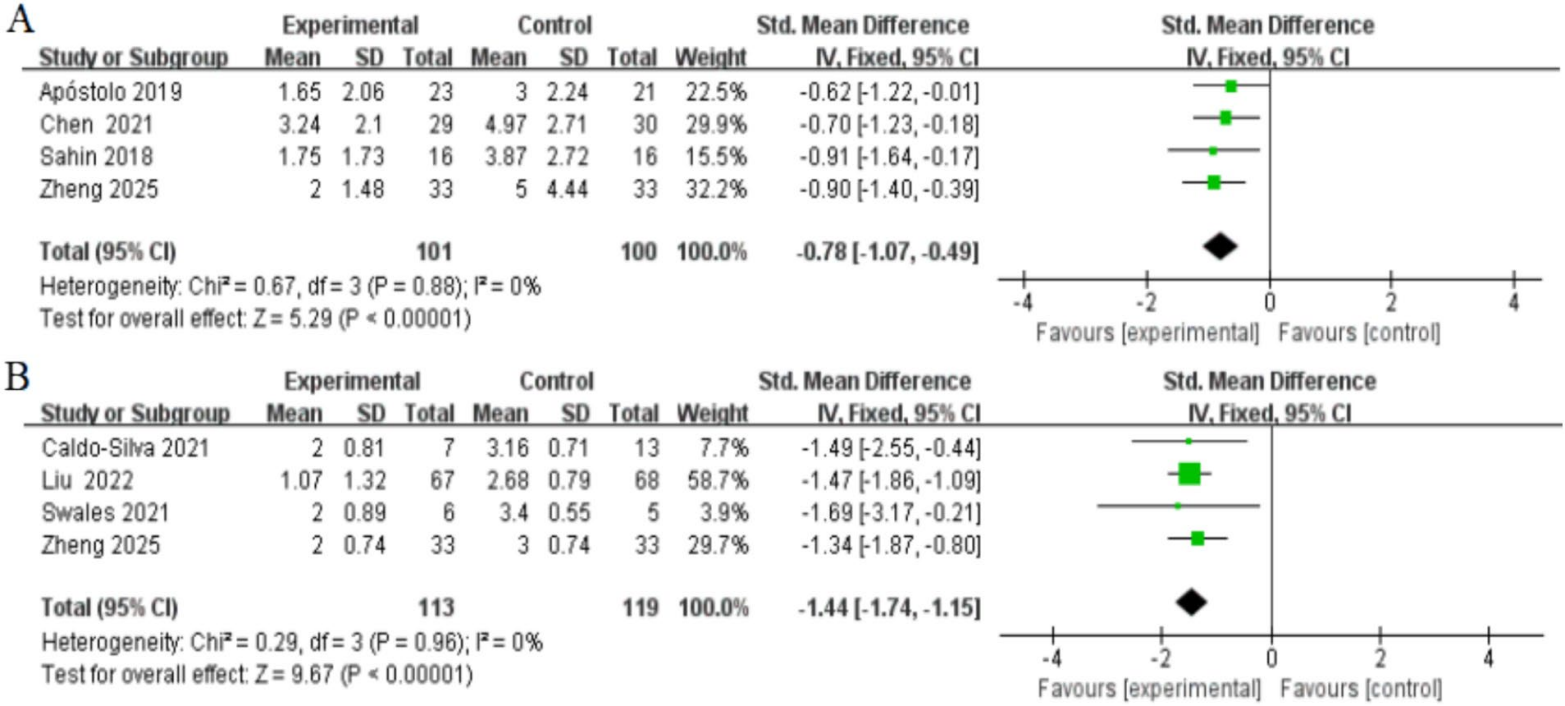
Forest plots of main outcomes. **(A)** Depressed; **(B)** frailty. Horizontal lines, 95% CIs of each study; green squares, SMDs of each study (size represents the weight given to the study in the meta-analysis): diamond, summary estimate; solid vertical line, null value.

Four studies, involving a total of 201 participants, assessed changes in depressive symptoms as measured by the GDS after the completion of the intervention. Two studies included both frail and pre-frail older adults (Chen et al., 2021; Zheng et al., 2025) while the other two studies focused solely on frail older adults (Apóstolo et al., 2019b; Sahin et al., 2018). Heterogeneity tests showed no significant between-study heterogeneity (*I*^2^ = 0%, *p* > 0.05), so fixed-effects models were used to combine effect sizes. The results showed that the exercise intervention significantly improved depressive symptoms in older adults [*SMD* = −0.78, 95% CI (−1.07, −0.49), *Z* = 5.29, *p* < 0.001] (Figure 5). In addition, Egger’s test did not detect significant publication bias (*p* > 0.05), as shown in Table 2.

#### 3.4.5 Frailty

Four studies, involving a total of 232 participants, were included to assess the effect of exercise interventions on frailty status in older adults. All studies assessed frailty status using the Frailty Phenotype score. Two studies included both frail and pre-frail older adults (Caldo-Silva et al., 2021; Zheng et al., 2025), while the other two studies focused solely on frail older adults (Liu et al., 2022; Swales et al., 2022). Heterogeneity tests showed no significant between-study heterogeneity (*I*^2^ = 0%, *p* > 0.05), so fixed-effects models were used to combine effect values. The results showed that the exercise intervention significantly improved frailty in older adults [*SMD* = −1.44, 95% CI (−1.74, −1.15), *Z* = 9.67, *p* < 0.001] (Figure 5). In addition, Egger’s test did not detect significant publication bias (*p* > 0.05), as shown in Table 2.

### 3.5 Subgroup analysis

The exercise intervention significantly improved physical functioning, quality of life of frail and cognitive functioning of older adults in nursing homes. To further explore the potential impact of participant characteristics and intervention characteristics on intervention effects, subgroup analyses were conducted. The results of the subgroup analyses showed that older adults aged ≤ 80 years had significantly better intervention effects than the >80 years group in terms of improved physical performance (*p* < 0.05) and quality of life (*p* < 0.001). Analyses for frequency of intervention showed that >2 interventions per week were better than 2 interventions per week in improving physical performance, lower limb strength, mobility and quality of life. Although the effect sizes of intervention cycles >12 weeks were higher than those of ≤12 weeks in terms of physical performance, grip strength, lower limb strength, and quality of life, the differences were not statistically significant. Detailed results of the subgroup analyses, including the effects of study participant characteristics (e.g., age) and intervention characteristics (e.g., intervention duration, frequency, and cycle) on physical performance, cognitive function, and quality of life, are detailed in Table 3.

**Table 3:** Subgroup analysis of study participant and intervention characteristics.

Variables	Physical performance				Mobility				Cognitive ability			
	*N*	*SMD* (95%CI)	*P* ^a^	*P* ^b^	*N*	*SMD* (95%CI)	*P* ^a^	*P* ^b^	*N*	*SMD* (95%CI)	*P* ^a^	*P* ^b^
Mean age				**0.04**				0.91				0.74
≤80	4	0.81 (0.50, 1.13)	<0.001		1	−6.36 (−9.28, −3.44)	<0.001		3	2.31 (1.27, 3.35)	<0.001	
>80	2	0.44 (0.26, 0.63)	<0.001		3	−6.06 (−10.80, −1.31)	<0.001		4	2.94 (−0.56, 6.44)	<0.05	
Minutes per session				0.11				<0.51				0.10
≤40	1	1.14 (0.38, 1.89)	0.003		2	−7.26 (−9.25, −5.27)	0.001		2	1.01 (−1.06, 3.08)	0.34	
>40	5	0.51 (0.35, 0.67)	<0.001		2	−5.16 (−11.13, 0.81)	0.009		5	2.79 (2.33, 3.24)	<0.001	
Times per week				**0.04**				**<0.001**				0.39
≤2	2	0.44 (0.26, 0.63)	<0.001		1	−2.11 (−2.91, −1.31)	<0.001		5	2.74 (2.29, 3.19)	<0.001	
>2	4	0.81 (0.50, 1.13)	<0.001		3	−8.10 (−8.75, −7.45)	<0.001		2	1.66 (−0.76, 4.09)	0.18	
Total weeks				0.85				0.18				0.96
≤12	4	0.53 (0.34, 0.72)	<0.001		3	−5.34 (−9.47, −1.20)	<0.001		5	0.65 (0.16, 1.14)	0.01	
>12	2	0.56 (0.27, 0.86)	<0.001		1	−8.20 (−8.89, −7.51)	<0.001		2	0.72 (−1.62, 3.05)	0.03	

Variables	Handgrip strength				Lower limb strength				Quality of life			
	*N*	*SMD* (95%CI)	*P* ^a^	*P* ^b^	*N*	*SMD* (95%CI)	*P* ^a^	*P* ^b^	*N*	*SMD* (95%CI)	*P* ^a^	*P* ^b^
Mean age				0.45				0.69				**<0.001**
≤80	2	0.94 (−0.09, 1.97)	0.07		2	−1.11 (−2.77, 0.56)	<0.001		1	5.00 (4.26, 5.74)	<0.001	
>80	2	3.22 (−2.65, 9.09)	0.28		4	−1.50 (−2.55, −0.46)	<0.001		3	0.95 (0.66, 1.24)	0.32	
Minutes per session				0.45				0.83			0.43	
≤40	2	0.94 (−0.09, 1.97)	0.07		2	−1.47 (−2.39, −0.55)	0.002		2	1.10 (0.78, 1.43)		
>40	2	3.22 (−2.65, 9.09)	0.28		4	−1.31 (−2.44, −0.18)	0.02		2	2.83 (−1.40, 7.06)		
Times per week				NA				**<0.001**				**<0.001**
≤2	0	NA	NA		3	−1.39 (−1.90, −0.88)	<0.001		1	5.00 (4.26, 5.74)	<0.001	
>2	4	2.00 (0.32, 3.68)	<0.001		3	−4.03 (−4.60, −3.47)	<0.001		3	0.95 (0.66, 1.24)	0.32	
Total weeks				0.40				0.78				0.27
≤12	2	0.85 (−0.38, 2.09)	0.18		3	−1.24 (−2.20, −0.28)	0.001		1	1.03 (0.28, 1.77)	<0.001	
>12	2	3.32 (−2.35, 8.99)	0.25		3	−1.50 (−2.98, −0.01)	<0.001		3	2.24 (0.21, 4.27)	<0.001	

a Subgroup studies.

b Differences between subgroups; 95% CI.

Bold values denote statistically significant differences (*p* < 0.05).

## 4 Discussion

The accelerating global trend of population aging is expected to significantly increase the number of frail elderly residents in nursing homes (Dent et al., 2023). This highlights the urgent need for cost-effective, nursing home-based interventions to enhance the physical and mental health of older adults. Exercise interventions are widely recognized as a key strategy for addressing frailty and functional impairments, as they not only reduce the prevalence of chronic diseases and risk of hospitalization but also delay functional decline (Casas-Herrero et al., 2019; Lim et al., 2024). While numerous clinical trials have shown that physical activity benefits older adults living in the community, such as by improving muscle strength, quality of life, and psychological and social well-being (Fairhall et al., 2012; Villareal et al., 2011) evidence on its effectiveness for nursing home residents remains limited.

To address this gap, we conducted a systematic review and meta-analysis to evaluate the effects of exercise interventions on physical functioning, cognitive functioning, and quality of life in nursing home residents. Additionally, we examined their impacts on frailty and depressive symptoms, and performed subgroup analyses to explore how participant characteristics and intervention attributes influence outcomes.

### 4.1 Effects of exercise interventions on physical functioning

This meta-analysis demonstrates that exercise interventions significantly improve physical function in frail or pre-frail older adults in nursing homes. The results are consistent with meta-analyses focusing on community-dwelling frail older adults, although the effect sizes observed here were more conservative, particularly for lower limb strength, mobility and physical function (Zhang et al., 2020). This may be attributed to differences in the definition of frailty, as the studies mentioned above defined frailty by significant declines in muscle strength, gait speed, or physical function, while this study also included pre-frail older adults. Exercise interventions may have more pronounced effects in older adults with physical disabilities or multiple chronic conditions. Although clinicians and researchers may be cautious in recommending and implementing exercise interventions for frail older adults, this group is likely to benefit significantly from such interventions (Zhang et al., 2020). For example, Chase et al. (2017) found that supervised physical activity interventions for frail older adults were particularly effective in improving balance.

Using a systematic review and meta-analysis, Peng et al. (2023) demonstrated that Otago exercise, based on moderate and consistent intensity principles, significantly improved the physical functioning of nursing home residents—a finding consistent with our results. Two studies in our meta-analysis utilized Otago exercise as the primary intervention, with García-Gollarte et al. (2023) reporting the largest effect sizes for mobility, grip strength, and lower limb strength. Resistance training emerged as a prominent component in six of the 16 studies included in our meta-analysis. Four studies explicitly employed low to moderate intensity resistance training protocols (i.e., <80% of one-repetition maximum [1-RM]), which have been shown to safely and effectively mitigate declines in muscle mass, strength, and physical function in older adults (Chodzko-Zajko et al., 2009; Lovering and Brooks, 2014). Consistent with this, a systematic review found that such protocols were equally effective in improving physical function, further supporting our findings (Borde et al., 2015). These results underscore the suitability of low-intensity physical activity for developing exercise intervention programs aimed at enhancing the physical function of frail elderly populations in nursing homes.

### 4.2 Effects of exercise interventions on cognitive function

Our findings indicate that exercise interventions can significantly improve cognitive function in frail or pre-frail elderly individuals in nursing homes. Previous meta-analyses suggested that resistance training is particularly effective for alleviating cognitive decline in dementia patients, whereas multi-component exercise is most effective therapy for preventing declines in cognitive and executive functions in individuals with mild cognitive impairment (Huang et al., 2022). In this study, mind–body exercises and multi-component exercises showed larger effects on cognitive function improvement in frail elderly individuals, while resistance training had a relatively smaller effect. Differences in outcomes across exercise types may be linked to participants’ impairment levels and the complexity of implementing interventions. Combining multiple exercise components may increase complexity, particularly for dementia patients with more severe impairment, making consistency between planning and execution difficult. For frail or pre-frail elderly individuals, multi-component and mind–body exercises appear to be effective types of exercise for improving cognitive function.

However, in practice, the effectiveness of multi-component exercise may be limited by several factors. For example, when performing multiple exercise components in sequence, it is challenging to ensure that each component meets its optimal duration and frequency, which may reduce its positive effects. In the two studies included in this meta-analysis (Apóstolo et al., 2019a; Ferreira et al., 2018), the exercise regimen of 2–3 sessions per week, each lasting 40 min, did not meet the recommended dosage by the American College of Sports Medicine (Piercy et al., 2018), and the improvement in cognitive function was relatively small. Frequency and duration are considered two key moderators of the effectiveness of multi-component exercise, with longer frequency and duration leading to greater effects (Bossers et al., 2015). Therefore, future research should consider these factors and further explore the impact of specific training programs on cognitive function using brain imaging techniques to enhance intervention outcomes. Moreover, given the small number of included studies and the variability in intervention methods and testing protocols, the interpretation of these findings should be cautious.

### 4.3 Effects of exercise interventions on quality of life

Our meta-analysis shows that exercise interventions significantly improve the quality of life of frail elderly individuals in nursing homes. A review included 16 studies that explored the impact of exercise interventions on the quality of life of healthy older adults (Raafs et al., 2020). The results indicated significant positive effects on both psychological and physical well-being, which aligns with our findings. Notably, Raafs et al. (2020) primarily focused on individual exercise programs, while our study included group-based activities such as Tai Chi, suggesting that group exercise may also enhance the quality of life in older adults. Group exercise helps increase social interaction, reduce depressive symptoms, and social engagement is an important component of quality of life (Komatsu et al., 2020). Furthermore, a meta-analysis by Zhang et al. (2020) of frail elderly individuals living in the community found that exercise interventions did not significantly improve their quality of life. This may be due to differences between nursing home and community environments: nursing homes typically provide more professional care, richer social activities, more systematic health management, and a safer living environment, all of which may contribute to improved quality of life. On the other hand, the QOL and WHOQOL-OLD scales used in our study provide a more comprehensive assessment of various aspects of quality of life, especially considering specific life circumstances, making the results more objective and holistic. We recommend that future research use standardized measurement tools and include more group-based exercise programs to more accurately estimate the impact of different types of exercise on the quality of life of older adults.

### 4.4 Effects of exercise interventions on frailty

Our meta-analysis demonstrates that exercise interventions significantly improve frailty syndrome in older adults. Previous studies have established a strong association between functional domains of physical performance and frailty syndrome, with muscle weakness being the most prevalent Fried frailty component in pre-frail populations. Improvements in physical functioning have been shown to mitigate the adverse effects of frailty in older adults (Angulo et al., 2020; Navarrete-Villanueva et al., 2021). Resistance exercise, widely used in clinical trials, has proven efficacy in enhancing both physical and physiological outcomes in frail older adults (Sahin et al., 2018; Swales et al., 2022). However, the three studies included in this meta-analysis incorporated not only resistance exercise but also multicomponent exercise programs and Otago exercise interventions. These findings highlight the potential value of diverse physical activity modalities in addressing frailty syndrome and suggest a broader applicability of tailored exercise interventions for this population.

### 4.5 Effects of exercise interventions on depression

Our meta-analysis indicates that exercise interventions significantly alleviate depressive symptoms in frail elderly individuals residing in nursing homes. The primary reason elderly individuals move into nursing homes is due to severe physical and mental health issues that prevent them from living independently, with over 40% of residents exhibiting depressive symptoms (Potter et al., 2018; Underwood et al., 2013). In addition, nursing home residents generally have low levels of physical activity, and their activity space—mainly limited to private rooms and adjacent living areas—may further worsen depressive symptoms (Jansen et al., 2017). Our findings suggest that higher levels of physical activity help older adults access meaningful locations and engage in social interactions, thereby alleviating depressive symptoms. Previous research also suggests that the benefits of exercise for mental health extend beyond reducing depression and anxiety, as exercise can enhance self-esteem through neurobiological mechanisms and generate positive effects (Apóstolo et al., 2019a). Therefore, there is sufficient evidence to urgently implement interventions aimed at encouraging elderly residents in nursing homes to engage in physical activities. It is also important to consider the potential impact of the physical environment in nursing homes on the mental and physical health of the elderly population. For example, a study conducted in the Netherlands found that bright lighting helped reduce depressive symptoms in residents of assisted living facilities (Riemersma-van der Lek et al., 2008).

### 4.6 Moderating effects of group characteristics and intervention characteristics

Subgroup analysis shows that age stratification and intervention characteristics (such as frequency, duration, and intervention cycle) significantly affect the physical function and quality of life of frail elderly individuals. Compared to those over 80 years old, participants aged ≤80 exhibited more significant improvements. This may be related to the primary characteristic of aging—decline in physical capacity, which is often associated with the loss of muscle mass, muscle contraction speed, and maximum strength. In individuals over 80, the prevalence of sarcopenia has exceeded 50% (Baumgartner et al., 1998; Fleg and Lakatta, 1988). Therefore, for the elderly population aged over 80, physical function may not be sufficient to support the completion of exercise plans, leading to lower compliance, which in turn limits the significant health benefits derived from exercise.

The U. S. Department of Health and Human Services recommends that effective exercise interventions for frail elderly individuals should combine balance training, strength training, and aerobic exercise, with at least 3 sessions per week, each lasting 30–45 min, over a period of 3–5 months (King et al., 2019). These recommendations align with the findings of this study—data show that exercise regimens with more than 2 sessions per week, each lasting ≥40 min, and an intervention duration of ≥12 weeks significantly improve the physical function and quality of life of nursing home residents. It is worth noting that, whether it is aerobic exercise or strength training, there is considerable variation in individuals’ responses to training (Fragala et al., 2019). Even when accounting for factors such as age, sex, and ethnicity, both responders and non-responders to training are apparent (Bouchard and Rankinen, 2001).

Therefore, exercise prescriptions for older adults should follow principles of personalization, periodization, and progression. Ideally, exercise and training programs should be professionally monitored and tailored to an individual’s unique physical, psychological, and medical challenges, addressing any comorbidities, orthopedic issues, activity limitations, or tolerance to different training modalities (such as endurance, strength, explosive power, or functional training) in order to gradually meet their evolving health and fitness goals. Additionally, it is crucial to consider the exercise motivations, preferences, and psychosocial factors of the elderly population. For example, interventions using group exercise to enhance social support, or setting realistic, progressive goals to increase self-efficacy, can significantly improve adherence to exercise plans (De Souto Barreto et al., 2016). From a practical perspective, the feasibility of implementing such exercise programs in nursing homes should also be taken into account, including considerations such as space availability, staff training, and fall-prevention strategies, which are essential to ensure both safety and effectiveness.

Future randomized controlled trials should build upon the existing dosage recommendations and, in line with the principles of individualized prescription, focus on identifying the optimal combinations of exercise modalities (e.g., aerobic, resistance, or balance training) and training parameters (e.g., 30 vs. 60 min per session, number of sessions per week) within institutional care settings. Such efforts will provide more precise, feasible, and evidence-based exercise guidelines for frail older adults in nursing homes.

### 4.7 Strengths and limitations

The main strength of this systematic review and meta-analysis is that it is the first time that a special group of frail elderly people in nursing homes was studied, and the significant effect of exercise intervention in improving their physical function, quality of life, frailty status and depressive symptoms was verified. This provides an important research basis for further exploring the optimal exercise mode and dosage effect. In addition, this study explored the intervention characteristics and participant characteristics in detail through subgroup analyses, which provides a scientific basis for the development and implementation of exercise intervention program for frail elderly in nursing homes.

This systematic review has several limitations. First, some outcome indicators in the included studies, such as quality of life and mobility, exhibited significant heterogeneity. (1) This may be attributed to differences in intervention protocols, such as variations in exercise type and intervention duration. (2) Different measurement tools were used to assess quality of life, each with distinct sensitivity and assessment focus. (3) Variability across nursing home settings, including the level of caregiver support and opportunities for social engagement, may also have contributed to the observed differences. These methodological and contextual factors should be carefully considered when interpreting the findings. Additionally, the Egger test was conducted with a relatively small number of included studies, warranting caution in interpreting the results. Second, the duration of the interventions was not systematically categorized, preventing an exploration of dose–response effects. The complexity of intervention types across the studies further complicates the identification of the most effective exercise modality. Finally, the subgroup analyses of mobility and quality of life included only three studies, which may affect the reliability and generalizability of these findings. In future studies targeting frail older adults in nursing homes, exercise interventions should not only take into account the institutional environment but also be tailored to individuals’ functional status, systematically exploring the most appropriate exercise type, duration, and frequency to develop prescription-based programs that maximize benefits.

## 5 Conclusion

This systematic review and meta-analysis address the current gap in research on exercise interventions for frail older adults in institutional settings and provide a theoretical foundation for optimizing exercise prescriptions. Specifically, exercise interventions have shown positive effects on physical function, mobility, grip strength, and lower limb strength. The findings also confirm that exercise has a beneficial impact on alleviating depressive symptoms and improving frailty status. Subgroup analyses further clarified the influence of participant characteristics (e.g., age) and intervention parameters (e.g., frequency, duration, and period) on key outcomes. However, the optimal type and dose of exercise for frail nursing home residents remain uncertain. This uncertainty arises from heterogeneity in intervention protocols, participant characteristics, outcome measures, and institutional conditions. These complexities highlight the need for future interventions to balance feasibility with clinical effectiveness and to be tailored to residents’ physical conditions and psychosocial needs.

In line with the findings of this study and the recommendations of the U. S. Department of Health and Human Services, exercise interventions for frail older adults in nursing homes should incorporate balance training (e.g., Tai Chi, Baduanjin) and strength training (e.g., resistance band or resistance exercises), delivered at least twice per week for approximately 45 min per session. Such programs may significantly improve health outcomes among institutionalized older adults. Health care providers and policymakers should regard exercise interventions as an essential component of frailty care and enhance feasibility and adherence through funding support, the development of standardized guidelines, and the implementation of individualized prescriptions.

## Data Availability

The original contributions presented in the study are included in the article/Supplementary material, further inquiries can be directed to the corresponding author.
