# Supplementary material for: Effects of exercise interventions on physical function, cognitive function and quality of life of frail older adults in nursing homes: a systematic review and meta-analysis
Source: Front Psychol. 2025 Sep 5;16:1679734. doi: 10.3389/fpsyg.2025.1679734 (PMC12447582; doi:10.3389/fpsyg.2025.1679734)
Supplement: Supplementary file 1 [file Table_1.DOCX]

**Supplementary Table 1**

| Supplementary Table 1: Detailed search string used in the electronic database PubMed |
| --- |
| ((((("Nursing Homes"[Mesh]) OR (((((((((((Nursing Homes[Title/Abstract]) OR (nursing center[Title/Abstract])) OR (homes nursing[Title/Abstract])) OR (care home[Title/Abstract])) OR (group home[Title/Abstract])) OR (rest home[Title/Abstract])) OR (retirement home[Title/Abstract])) OR (residential[Title/Abstract])) OR (facility[Title/Abstract])) OR (institution[Title/Abstract])) OR (Homes for the Aged[Title/Abstract]))) AND (("Frail Elderly"[Mesh]) OR ((((((((((((((((((frail[Title/Abstract]) OR (prefrail[Title/Abstract])) OR (pre frail[Title/Abstract])) OR (pre frail[Title/Abstract])) OR (Frailties[Title/Abstract])) OR (Frailness[Title/Abstract])) OR (Frail Elderly[Title/Abstract])) OR (Elderly, Frail[Title/Abstract])) OR (Frail Elders[Title/Abstract])) OR (Elder, Frail[Title/Abstract])) OR (Elders, Frail[Title/Abstract])) OR (Frail Elder[Title/Abstract])) OR (Frail Older Adults[Title/Abstract])) OR (Adult, Frail Older[Title/Abstract])) OR (Adults, Frail Older[Title/Abstract])) OR (Frail Older Adult[Title/Abstract])) OR (Older Adult, Frail[Title/Abstract])) OR (Older Adults, Frail[Title/Abstract])))) AND (("Aged"[Mesh]) OR (((((((aged[Title/Abstract]) OR (aging[Title/Abstract])) OR (ageing[Title/Abstract])) OR (elder[Title/Abstract])) OR (oldest old[Title/Abstract])) OR (old[Title/Abstract])) OR (senior[Title/Abstract])))) AND (((((((((((((((((((Exercise[MeSH Terms]) OR (Motor Activity[MeSH Terms])) OR (Movement[MeSH Terms])) OR (Exercise Therapy[MeSH Terms])) OR (Motion Therapy, Continuous Passive[MeSH Terms])) OR (Exercise Movement Techniques[MeSH Terms])) OR (Physical Exertion[MeSH Terms])) OR (Exercise Tolerance[MeSH Terms])) OR (Endurance Training[MeSH Terms])) OR (Resistance Training[MeSH Terms])) OR (Weight Lifting[MeSH Terms])) OR (Circuit-Based Exercise[MeSH Terms])) OR (Vibration[MeSH Terms])) OR (("Exercise"[Title/Abstract] OR "Exercises"[Title/Abstract] OR "Physical Activity"[Title/Abstract] OR "Physical Activities"[Title/Abstract] OR "Activity, Physical"[Title/Abstract] OR "Activities, Physical"[Title/Abstract] OR "Physical Exercise"[Title/Abstract] OR "Physical Exercises"[Title/Abstract]))) OR (("Acute Exercise"[Title/Abstract] OR "Acute Exercises"[Title/Abstract] OR "Exercise, Acute"[Title/Abstract] OR "Exercises, Acute"[Title/Abstract] OR "Exercise, Isometric"[Title/Abstract] OR "Exercises, Isometric"[Title/Abstract] OR "Isometric Exercise"[Title/Abstract] OR "Isometric Exercises"[Title/Abstract] OR "Exercise, Aerobic"[Title/Abstract] OR "Aerobic Exercise"[Title/Abstract] OR "Aerobic Exercises"[Title/Abstract] OR "Exercises, Aerobic"[Title/Abstract]))) OR (("Exercise Training"[Title/Abstract] OR "Exercise Trainings"[Title/Abstract] OR "Training, Exercise"[Title/Abstract] OR "Exercise Therapy"[Title/Abstract] OR "Remedial Exercise"[Title/Abstract] OR "Rehabilitation Exercise"[Title/Abstract] OR "Rehabilitation Exercises"[Title/Abstract] OR "Exercise, Rehabilitation"[Title/Abstract] OR "Exercise method"[Title/Abstract] OR "exercise prescription"[Title/Abstract]))) OR (("Activity"[Title/Abstract] OR "Activities"[Title/Abstract] OR "Motor Activity"[Title/Abstract] OR "Motor Activities"[Title/Abstract] OR "Sport"[Title/Abstract] OR "Sports"[Title/Abstract] OR "Movement"[Title/Abstract] OR "Movements"[Title/Abstract]))) OR (("aerobic training"[Title/Abstract] OR "cardio training"[Title/Abstract] OR "endurance exercise"[Title/Abstract] OR "Exercise Tolerance"[Title/Abstract] OR "Endurance Training"[Title/Abstract] OR "Resistance Training"[Title/Abstract] OR "Strength Training"[Title/Abstract] OR "Weight-Bearing Strengthening Program"[Title/Abstract] OR "strength training"[Title/Abstract] OR "strength exercise"[Title/Abstract] OR "Weight Lifting"[Title/Abstract] OR "Balance training"[Title/Abstract] OR "mixed exercises"[Title/Abstract] OR "combined exercises"[Title/Abstract] OR "Multimodal exercise"[Title/Abstract]))) OR (("Vibration"[Title/Abstract] OR "Vibrations"[Title/Abstract] OR "vibration training"[Title/Abstract] OR "local muscle vibration"[Title/Abstract] OR "Whole body vibration"[Title/Abstract] OR "WBV"[Title/Abstract] OR "whole body vibration exercise"[Title/Abstract] OR "WBVE"[Title/Abstract] OR "Vibration therapy"[Title/Abstract] OR "vibrational therapy"[Title/Abstract] OR "vibration treatment"[Title/Abstract] OR "vibrotherapeutics"[Title/Abstract] OR "Vibrotactile"[Title/Abstract] OR "Vibration plate"[Title/Abstract] OR "vibrating platform"[Title/Abstract] OR "oscillating platforms"[Title/Abstract])))) AND ((((((((Randomized Controlled Trials as Topic[MeSH Terms]) OR (Controlled Clinical Trials as Topic)) OR (Clinical Trials as Topic[MeSH Terms])) OR (Controlled Clinical Trials as Topic[MeSH Terms])) OR (Intention to Treat Analysis[MeSH Terms])) OR (Single-Blind Method[MeSH Terms])) OR (Double-Blind Method[MeSH Terms])) OR ((((((((((Randomized Controlled Trial[Publication Type]) undefined (Controlled Clinical Trial[Publication Type])) undefined (Clinical Trial[Publication Type])) OR (random*[Text Word])) OR (allocation[Text Word])) OR (random allocation[Text Word])) OR (single blind[Text Word])) OR (double blind[Text Word])) OR (randomized controlled trial[Text Word])) OR (RCT[Text Word]))) |

Note: Using PubMed as an example, the rest of the databases were searched based on the above vocabulary conversion format.
